# Analysis of the Effect of ^60^Co-*γ* Irradiation Sterilization Technology on the Chemical Composition of Saffron Using UPLC and UPLC/Q-TOF-MS

**DOI:** 10.1155/2018/2402676

**Published:** 2018-03-04

**Authors:** Ding-qiang Luo, Shan-shan Zhao, Yu-rong Tang, Qing-jun Wang, Hai-jing Liu, Shuang-cheng Ma

**Affiliations:** ^1^Shaanxi Institute for Food and Drug Control, Xi'an, China; ^2^Shaanxi University of Chinese Medicine, Xianyang, China; ^3^National Institutes for Food and Drug Control, Beijing, China

## Abstract

To evaluate the effect of ^60^Co-γ irradiation sterilization technology on the chemical composition of saffron, we collected 10 batches of saffron samples and treated them with different irradiation doses. UPLC characteristic chromatogram showed that there was no significant effect of irradiation on 13 common peak areas. The results of cluster analysis and principal component analysis showed that there were no differences in the chemical composition in nonirradiated and irradiated samples. UPLC/Q-TOF-MS identified 40 characteristic components of saffron, and the results showed that all of these were detected in the saffron samples both with and without irradiation. Irradiation doses at or below 10 kGy had no significant effect on the chemical components of saffron. This provides a sound basis for the use of ^60^Co-γ ray irradiation sterilization technology during the preparation of original powder saffron as a medicinal herb, for the effective destruction of mycotoxin contamination.

## 1. Introduction

Mold is a major cause of deterioration during the harvest and storage of food and medicinal plants. It is relatively easy to avoid this type of contamination because molds can be easily seen on such products. However, contamination with mycotoxins, the toxic metabolite products of molds, is hidden because of the microscopic size of the particles concerned. When food is contaminated with mycotoxins, the sensory characteristics of the food generally do not change significantly, so contamination can easily pass unnoticed. Furthermore, mycotoxins are heat resistant; therefore, cooking is not an effective method for their removal [1]. According to the published literature, mycotoxins have been shown to be carcinogenic [2], mutagenic, teratogenic, and toxic to cells [3]. Effect of irradiation on chemical composition and some other aspects of some medicinal herbs has been studied [4–9]. However, the specific application of irradiation sterilization technology on medicinal herbs needs to be considered based on the characteristics of each species.

Cobalt 60 (^60^Co-*γ*) irradiation technology is an effective method for the decontamination of food and medicines from both insect infestation and mycotoxin contamination. It works by destroying the microbes in a sample and by damaging the DNA material in living organisms. Under the correct conditions, gamma irradiation can effectively destroy mycotoxin, so it is an important technology for plants decontamination [10]. To date, the Codex Alimentarius Commission (CAC) has permitted the use of irradiation treatment for the decontamination of plant-derived food materials in more than 55 countries including the United States, the European Union, South Korea, and China. An irradiation dose below 10 kGy is permitted in this context [11–13].

Saffron (Iridaceae) reportedly has certain positive health effects, such as activating blood flow and removing blood stasis, cooling the blood detoxification, removing depression, and anchoring the mind [14]. It is widely used to promote good blood circulation, to relieve blood stasis [15] and to treat cardiovascular disease [16]. In Chinese medicinal preparations, saffron is often used in powder form (such as in Ershiwuwei Coral pills, Ershiwuwei Songshi pills, and Dingkun pills). Irradiation is widely used in the manufacture of traditional Chinese medicines for the control of mycotoxin contamination, including in saffron preparations. However, its effect on the quality and chemical composition of saffron is not well understood and demands research. Besides, chemometric methods were often employed to identify differences between batches [17–19]. In this study, we collected 10 batches of saffron samples and treated them with different irradiation doses to evaluate the effect on chemical composition of the samples. We used UPLC coupled with cluster analysis and principal component analysis (PCA) to evaluate the results qualitatively and semiquantitatively. UPLC/Q-TOF-MS was used for the identification of the chemical characteristic components of the saffron samples with and without irradiation.

## 2. Experimental

### 2.1. Samples

Ten batches of saffron samples were purchased between 2014 and 2015, from Qinghai (numbers 1, 2, 3, 4, 5, 6, 7, 8, 9, and 10). The samples were identified to be dried stigma of *Crocus sativus* L (Iridaceae) by Shuangcheng Ma, the researcher at National Institutes for Food and Drug Control.

### 2.2. Chemicals

Acetonitrile (HPLC grade) and methanol (HPLC grade) were purchased from Merck (Merck Sharp & Dohme Corp.). Distilled water was purchased from Watsons Water (Guangzhou Watsons Group Co., Ltd., China). For chemical analysis, pure phosphoric acid, glacial acetic acid, and ethanol were bought from the National Pharmaceutical Group Chemical Reagent Co., Ltd. (China). The following reference standards were provided by the National Institutes for Food and Drug Control: Crocin I (111588-200501) and Crocin II (111589-200802).

### 2.3. Sample Preparation

For each sample to be analyzed, 200 mg of the powder of saffron was accurately weighed and mixed with 50 ml of 60% methanol solution. After weighing the resulting solution, it was subjected to ultrasonic treatment for 30 min (at a power of 250 W, frequency of 40 kHz, and temperature of 25°C), cooled, and then reweighed. Make up the less weight with methanol. After filtration, the primary filtrate was discarded and the secondary filtrate obtained from filtration through a 0.22 *μ*m microporous membrane was retained for chromatography analysis.

### 2.4. Reference Standard Preparation

10.21 mg Crocin I was accurately weighed and placed in a 100 ml volumetric flask, dissolved in methanol and diluted to volume. From this, 2 ml was transferred into a 10 ml volumetric flask and diluted with methanol to volume. The flask was shaken, and the solution was then filtered through a 0.22 *μ*m microporous membrane. The resulting reference standard solution was 20.42 *μ*g/ml in concentration.

5.63 mg Crocin II was accurately weighed and placed in a 100 ml volumetric flask, dissolved in methanol and diluted to volume. From this, 3 ml was transferred into a 10 ml volumetric flask and diluted with methanol to volume. The resulting reference standard solution was 16.89 *μ*g/ml in concentration.

### 2.5. Irradiation

The saffron samples were packaged in plastic bottles. Subsamples of all 10 saffron samples were analyzed chromatographically without irradiation (control group), followed by analysis of 10 further subsamples after being exposed to ^60^Co-*γ* irradiation doses of 10 kGy for 5 h or 10 kGy for 10 h in Xi'an Beilin Pharmaceutical Co., Ltd. (China). The absorbed dose was determined with a silver dichromate dosimeter.

## 3. Equipment

The UPLC method utilized an Agilent EC-C18 column (4.6 mm × 150 mm, 2.7 *μ*m). The mobile phases consisted of solvent A (methanol) and solvent B (0.5% phosphoric acid in water, v/v). The optimized UPLC elution conditions were as follows: 0–5 min, 10-10% A; 5–10 min, 10–30% A; 10–50 min, 30–40% A; 50–65 min, 40–50% A; 65–90 min, 50–70% A; 90–100 min, 70–80% A; 100–101 min, 80–10% A; and 101–110 min, 10-10% A. The flow rate was set at 1.0 ml/min. The injection volume was 5 *μ*l. The column was maintained at 30°C. The detection wavelength was 312 nm.

The UPLC/Q-TOF-MS method utilized an Agilent EC-C18 column (4.6 mm × 150 mm, 2.7 *μ*m). The mobile phases consisted of solvent A (0.1% glacial acetic acid in water, v/v) and solvent B (acetonitrile). The optimized elution conditions were as follows: 0–5 min, 10-10% B; 5–10 min, 10–30% B; 10–50 min, 30–40% B; 50–65 min, 40–50% B; 65–90 min, 50–70% B; 90–100 min, 70–80% B; 100–101 min, 80–10% B; and 101–110 min, 10–10% B. The flow rate was set at 0.3 ml/min, and the column was maintained at 30°C. The injection volume was 2 *μ*l. The detection wavelength was 254 nm.

The mass spectrometry conditions were as follows: electron spray ionization (ESI) in negative ion mode; fragmentor voltage 130 V; drying gas nitrogen 10 L/min; nebulizer 45 psi; gas temperature 350°C; skimmer voltage 45 V; OCT 1RF Vpp 750 V; scan range 100–1100; and acquisition rate time 2/s.

## 4. Data Analysis

Paired tests were performed to compare the chromatography peak areas (of the two common peaks) with and without irradiation of the samples. The identity and composition of the individual chemical components in the saffron samples were determined from the chromatography results, with and without irradiation.

Using the characteristic peak area data produced by the UPLC analyses, PCA was conducted to distinguish between the saffron samples according to their treatment (irradiated versus nonirradiated). Unsupervised PCA was conducted with the peak area of each identified chemical component as the *X* variable and with batch number as the *Y* variable.

The difference between the chemical composition of the nonirradiated and irradiated samples was further identified and analyzed by HCA using SPSS version 20.0 software (SPSS, Inc., Chicago, IL). The 13 characteristic common peak areas and the sample size of different groups (the nonirradiated, irradiated, and different irradiation doses of saffron) were used as parameters. The clustering relationship between each sample was calculated using a 33-row and 15-column matrix that was built by SPSS 18.0 statistical software.

## 5. Results and Discussion

### 5.1. Chemical Differences between Nonirradiated and Irradiated Saffron Samples

**Figure 1:**
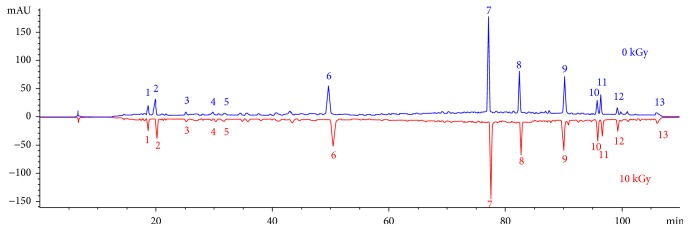
UPLC feature chromatograms of nonirradiated and irradiated saffron.

**Figure 2:**
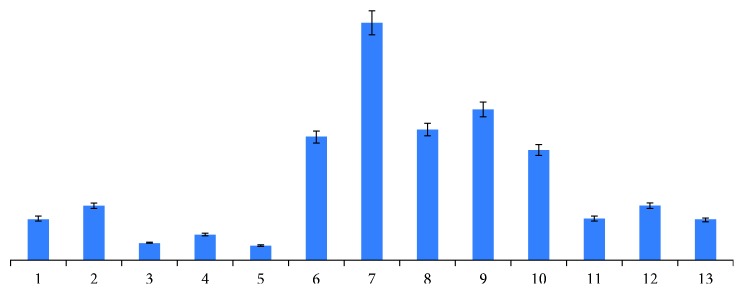
Comparison of mean difference of common peaks.

The qualitative and semiquantitative comparison of the chemical composition of nonirradiated and irradiated saffron was made through UPLC and Q-TOF-MS analysis. A total of 13 characteristic chromatographic peaks were detected from 10 batches of saffron (Figure 1, Supplementary Figure 1). Peak number 8 was Crocin I. The relative retention time of the characteristic chromatograms produced by the saffron samples was calculated with peak number 8 as the standard peak (S peak) (Supplementary Table 1). The average difference in the common peak area produced by each saffron sample with and without irradiation was calculated (Figure 2). This showed that peaks 6, 7, 8, 9, and 10 all produced a certain degree of reduction after irradiation, with peak number 7 producing more marked decreases than the other peaks. The relative deviations were less than 10% before and after irradiation. The data were tested by paired *t* test, and bilateral sig value of *t* test was greater than 0.01. This result suggests that *γ*-irradiation did not have a significant effect on the chemical component composition of the saffron samples.

### 5.2. HCA and PCA Analysis

**Figure 3:**
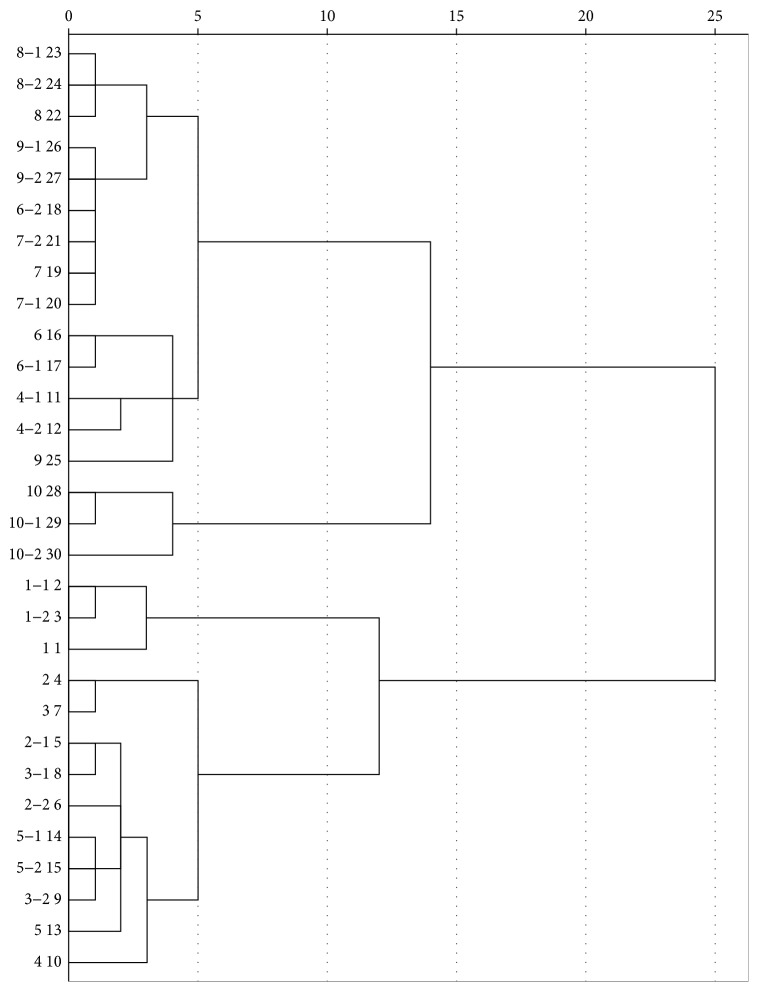
Hierarchical cluster analysis (HCA) of nonirradiated and irradiated saffron. *Note. n*: nonirradiation group; *n* -1: 10 kGy irradiation for 5 h; *n* -2: 10 kGy irradiation for 10 h (*n* = 1–10, number of the 10 batches of samples).

HCA and PCA were used to quantitatively evaluate the diversity of the chemical component differences between the nonirradiated and irradiated plant samples. In HCA, we used the monitoring method to reveal and compare the connection distance between “different” and “similar” samples, after data normalization processing. In Figure 3, the data relating to the different irradiation dose levels (control “nonirradiated”/10 kGy for 5 h/10 kGy for 10 h) from the same batch of saffron were gathered together to form one group. Under this analysis, nonirradiated, irradiated, and even different irradiation doses of the same batch of sample were found to be very close, indicating that the difference produced by the different irradiation doses was small. There was no significant effect of the irradiation dose on the chemical composition of the saffron samples.

**Figure 4:**
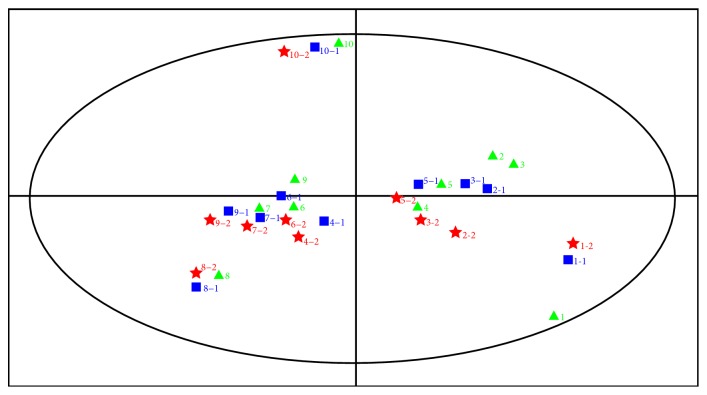
Principal component analysis (PCA) of nonirradiated and irradiated saffron. *Note*. *n*: nonirradiation group; *n *-1 10 kGy irradiation for 5 h; *n *-2 10 kGy irradiation for 10 h (*n* = 1–10, number of the 10 batches of samples).

PCA, as an unsupervised pattern recognition method, distinguished the degree of difference between the irradiated and nonirradiated samples. The data are processed by dimension reduction to carry on PCA, and display of the score and the load for the samples is obtained, with the peak area of each component as the *X* variable and the saffron batch number as the *Y* variable. The 30 sets of chromatography data from the saffron samples that had been subjected to the 3 different irradiation doses (10 samples per dose) were analyzed by PCA. The degree to which the first two variables explained the total variance among the samples was 93.4%, indicating that the group of saffron samples was suitable for analysis by PCA. From the loading diagrams for each sample, it was observed that the “distance” between saffron samples from the same batch that had received different irradiation treatments was closer than that between saffron samples from different batches. The different treatment groups could not be easily distinguished. This is consistent with the results of the hierarchical cluster analysis, which indicated that there was no significant effect of irradiation treatment on the composition of chemical components in the saffron samples (Figure 4).

### 5.3. Q-TOF-MS Chemical Composition Identification

In order to further determine the effects of irradiation on the chemical composition of saffron, UPLC-ESI-Q-TOF-MS was used to analyze the differences of Crocin I, II, III, and other characteristic chemical composition between with and without irradiation. The results showed that certain characteristic components including Crocin I, II, III, and other components were all detected in both irradiated and nonirradiated samples. The results can be seen in Table 1 (Supplementary Table 2 and Figure 2). Through the analysis of the mass spectromety fragments, coupled with determination of their accurate molecular weight, as well as their comparison with data from the relevant reference standards, the characteristic components of the saffron samples were identified. A total of 40 components were detected in the saffron samples, and these were present both with and without irradiation. These results indicate that irradiation at the specified doses had no significant effect on the characteristic components of the saffron samples.

Component 23 *m*/*z* is 346.1628 [M-H]^−^ and is identified as picrocrocinic acid (C_16_H_26_O_8_) compared with the literature. Peak 30 (*t*_*R*_ = 11.929 min, *m*/*z* = 330.3678) was identified as picrocrocin (C_16_H_26_O_7_). Peak 36 (*t*_*R*_ = 64.023 min, *m*/*z* = 975.9746) coincided with Crocin I and was identified as Crocin I (C_44_H_46_O_24_). Peak 37 (*t*_*R*_ = 68.970 min, *m*/*z* = 813.9245) coincided with Crocin II and was identified as Crocin II. Peak 40 *m*/*z* is 652.2731 and is identified as Crocin III (C_32_H_44_O_14_) according to the literature [20].

## 6. Conclusion

This study evaluated the effect of ^60^Co-*γ* irradiation on the chemical composition of saffron. Two doses of irradiation were tested, up to a maximum of 10 kGy (for 10 h). A variety of statistical methods, including paired *t*-tests to compare chromatographic peak areas and PCA and HCA, were used to determine differences between the irradiated and nonirradiated samples. The results indicated that irradiation had no significant effect on the chemical components of saffron. However, with the increase of irradiation dose and time, there is a trend that the peak area is decreased, so the irradiation dose and time should be strictly controlled. These findings provide a sound technical basis for the irradiation of saffron medicinal herb using original powder using ^60^Co-*γ* for the effective destruction of mycotoxin contamination and indicate that irradiation technology has good application prospects in the sterilization of saffron and other medicinal plants.

## Figures and Tables

**Table 1:** Chemical components identified from nonirradiated and irradiated saffron.

Peak number	RT	Experimental (*m*/*z*)	Calculated (*m*/*z*)	Difference (ppm)	MS/MS fragments	Formula	Proposed compounds	0 kGy	10 kGy
1	0.74	194.079	194.079	1.26	112.9858, 176.9345	C_7_H_14_O_6_	Beta-methyl-D-glucoside	√	√
2	0.756	120.0423	120.0423	2.43	616.6543	C_4_H_8_O_4_	Methyl allyl tetrasulfide	√	√
3	0.756	180.0634	180.0634	1.21	124.0433, 400.5046, 977.3344	C_6_H_12_O_6_	Cocositol	√	√
4	0.831	342.1162	342.1162	1.1	245.0399, 1064.3185	C_12_H_22_O_11_	Cellobiose	√	√
5	0.856	150.0528	150.0528	1.48	426.1106, 740.8576	C_5_H_10_O_5_	Apiose	√	√
6	0.856	278.1002	278.1002	2.32	101.0246, 197.3849	C_11_H_18_O_8_	Tuliposide A	√	√
7	1.038	150.0528	150.0528	1.2	968.8609, 1055.9846	C_5_H_10_O_5_	Apiose	√	√
8	1.038	196.0583	196.0583	0.93	368.0468	C_6_H_12_O_7_	Gluconic acid	√	√
9	1.08	76.016	76.016	0.99	112.9856, 395.2799, 1095.0428	C_2_H_4_O_3_	Glycolic acid	√	√
10	1.08	90.0317	90.0317	0.82	125.3414	C_3_H_6_O_3_	Dihydroxyacetone	√	√
11	1.329	46.0055	46.0055	1.6	1635.3649	CH_2_O_2_	Formic acid	√	√
12	1.329	106.0266	106.0266	1.3	567.0849, 1227.0691	C_3_H_6_O_4_	D-glyceric acid	√	√
13	1.404	210.074	210.074	1.21	721.5432	C_7_H_14_O_7_	Coriose	√	√

## Supplementary Materials

Supplementary 1 Figure S1: feature graph of 10 batches specimens of saffron.

Supplementary 2 Table S1: relative retention time feature graph of saffron.

Supplementary 3 Table S2: chemical components identified from nonirradiated and irradiated saffron.

Supplementary 4 Figure S2: UPLC/Q-TOF-MS EIC (extract ion chromatograms) of saffron.
