# Supplementary figures and images for: Analysis of the Effect of 60Co-γ Irradiation Sterilization Technology on the Chemical Composition of Saffron Using UPLC and UPLC/Q-TOF-MS
Source: J Anal Methods Chem. 2018 Mar 4;2018:2402676. doi: 10.1155/2018/2402676 (PMC5857329; doi:10.1155/2018/2402676)

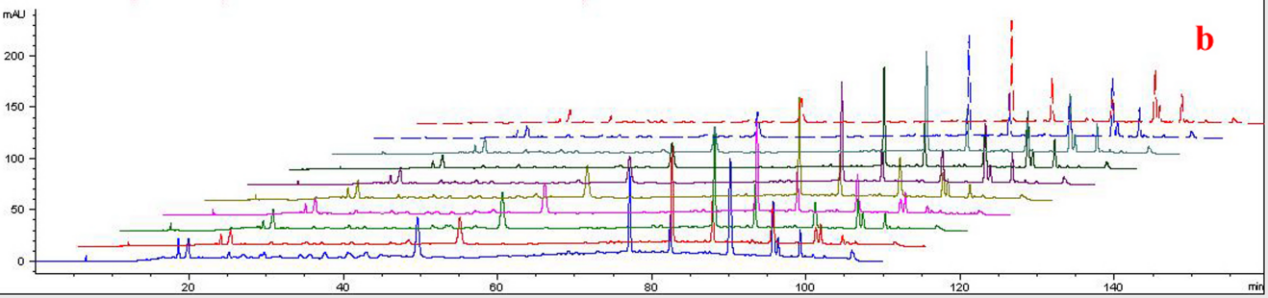


Feature graph of 10 batches specimens of saffron

Supplement: Supplementary 1 — Figure S1: feature graph of 10 batches specimens of saffron. [file 2402676.f1.docx]

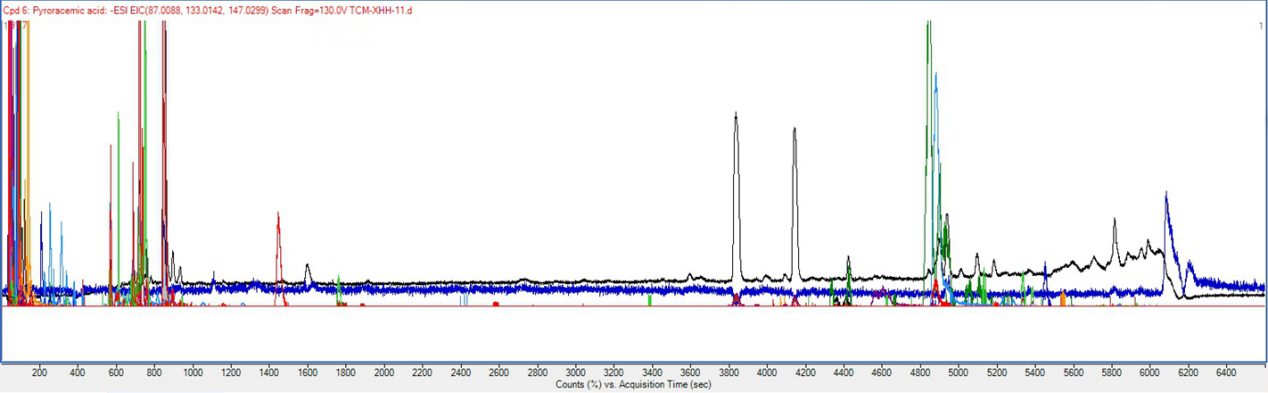


UPLC/Q-TOF-MS EIC(extract ion chromatograms) of saffron

Supplement: Supplementary 4 — Figure S2: UPLC/Q-TOF-MS EIC (extract ion chromatograms) of saffron. [file 2402676.f3.docx]
