# Supplementary material for: Analysis of the Effect of 60Co-γ Irradiation Sterilization Technology on the Chemical Composition of Saffron Using UPLC and UPLC/Q-TOF-MS
Source: J Anal Methods Chem. 2018 Mar 4;2018:2402676. doi: 10.1155/2018/2402676 (PMC5857329; doi:10.1155/2018/2402676)
Supplement: Supplementary 2 — Table S1: relative retention time feature graph of saffron. [file 2402676.f2.pdf]

3. TABLE 1: Relateive retention time feature graph of saffron

Sample	Peak 1	Peak 2	Peak 3	Peak 4	Peak 5	Peak 6	Peak 7	Peak 8 (S)	Peak 9	Peak 10	Peak 11	Peak 12	Peak 13
1	0.225	0.241	0.305	0.362	0.385	0.605	0.937	1.000	1.095	1.162	1.170	1.205	1.286
2	0.225	0.241	0.305	0.361	0.385	0.603	0.936	1.000	1.095	1.163	1.170	1.205	1.286
3	0.225	0.241	0.305	0.361	0.385	0.602	0.936	1.000	1.095	1.163	1.170	1.205	1.286
4	0.225	0.241	0.305	0.361	0.385	0.602	0.936	1.000	1.095	1.163	1.170	1.205	1.286
5	0.225	0.241	0.305	0.360	0.385	0.602	0.936	1.000	1.095	1.162	1.168	1.205	1.286
6	0.225	0.241	0.305	0.360	0.385	0.602	0.936	1.000	1.095	1.162	1.170	1.205	1.286
7	0.225	0.241	0.305	0.360	0.385	0.602	0.936	1.000	1.095	1.162	1.170	1.205	1.286
8	0.225	0.241	0.305	0.361	0.385	0.602	0.936	1.000	1.095	1.162	1.170	1.205	1.286
9	0.225	0.241	0.305	0.360	0.385	0.602	0.936	1.000	1.095	1.162	1.170	1.205	1.286

10	0.225	0.241	0.305	0.360	0.385	0.602	0.936	1.000	1.095	1.162	1.170	1.205	1.286

4.

TABLE 2: Chemical components identified from non-irradiaated and irradiated saffron

peak no.	RT	Experimental m/z	calculated m/z	Diff (ppm)	MS/MS Fragments	Formula	Proposed compounds	0 kGy	10 kGy
1	0.74	194.079	194.079	1.26	112.9858,176.9345	C7 H14 O6	beta-Methyl-D-glucoside	√	√
2	0.756	120.0423	120.0423	2.43	616.6543	C4 H8 O4	Methyl allyl tetrasulfide	√	√
3	0.756	180.0634	180.0634	1.21	124.0433,400.5046,977.3344	C6 H12 O6	Cocositol	√	√
4	0.831	342.1162	342.1162	1.1	245.0399,1064.3185	C12 H22 O11	Cellobiose	√	√
5	0.856	150.0528	150.0528	1.48	426.1106,740.8576	C5 H10 O5	Apiose	√	√
6	0.856	278.1002	278.1002	2.32	101.0246,197.3849	C11 H18 O8	Tuliposide A	√	√
7	1.038	150.0528	150.0528	1.2	968.8609,1055.9846	C5 H10 O5	Apiose	√	√
8	1.038	196.0583	196.0583	0.93	368.0468	C6 H12 O7	Gluconic acid	√	√
9	1.08	76.016	76.016	0.99	112.9856,395.2799,1095.0428	C2 H4 O3	Glycolic acid	√	√
10	1.08	90.0317	90.0317	0.82	125.3414	C3 H6 O3	Dihydroxyacetone	√	√
11	1.329	46.0055	46.0055	1.6	1635.3649	C H2 O2	Formic acid	√	√
12	1.329	106.0266	106.0266	1.3	567.0849,1227.0691	C3 H6 O4	D-Glyceric acid	√	√
13	1.404	210.074	210.074	1.21	721.5432	C7 H14 O7	Coriose	√	√
14	1.404	164.0685	164.0685	1.81	317.2131,1079.9937	C6 H12 O5	2-O-Methyl-D-xylose	√	√
15	1.504	150.0164	150.0164	0.85	259.7354,393.0590	C4 H6 O6	Tartaric acid	√	√
16	1.504	210.0376	210.0376	0.61	461.7537,1195.5139	C6 H10 O8	Mucic acid	√	√
17	1.529	88.016	88.016	0.74	248.1214,647.9147,1461.2433	C3 H4 O3	Pyroracemic acid	√	√
18	1.529	134.0215	134.0215	0.56	213.4008,324.1017,368.3939	C4 H6 O5	Malic acid	√	√

19	1.637	194.0426	194.0426	1.13	1161.9264	C6 H10 O7	Glucuronic acid	√	√
20	2.326	132.0059	132.0059	1.49	115.0047	C4 H4 O5	Oxalacetic acid	√	√
21	2.326	192.027	192.027	1.02	176.9365,532.1482	C6 H8 O7	Citric acid	√	√
22	9.454	184.1099	184.1099	2.35		C10 H16 O3	Elsholtzidiol	√	√
23	9.603	346.1628	346.1628	1.38	154.9723,247.8580,327.8214	C16 H26 O8	Picrocrocinic acid	√	√
24	10.276	478.1686	478.1686	0.92	322.8507,424.7964	C20 H30 O13	2,4,6-Trimethoxyphenol-1-O-beta-D-apiofuranosyl-(1-->6)-beta-D-glucopyranoside	√	√
25	10.276	432.1632	432.1632	0.63	612.9297,336.8868	C19 H28 O11	2-C-Methyl-D-erythritol 1-O-beta-D-(6-O-4-methoxybenzoyl)glucopyranoside	√	√
26	10.276	492.1843	492.1843	1.02	322.8509,440.7707	C21 H32 O13	Cistanoside E	√	√
27	10.592	136.0524	136.0524	1.2	171.5367,236.0642,646.6670	C8 H8 O2	4-Methyl salicylaldehyde	√	√
28	10.592	150.0681	150.0681	1.08	816.3961,1046.0708,1370.9614	C9 H10 O2	3,4-Dimethylbenzoic acid	√	√
29	10.592	196.0736	196.0736	0.83	512.2025,1641.8895	C10 H12 O4	2,4-Dihydroxy-6-methoxy-3-methylacetophenone	√	√
30	11.929	330.3678	330.3678	1.33	144.0295,199.3524,232.9037	C16 H26 O7	Picrocrocin	√	√
31	12.502	342.1315	342.1315	-0.34	180.0788	C16 H22 O8	Baihuaqianhuoside	√	√
32	12.502	356.1471	356.1471	0.42	322.8457,415.7080	C17 H24 O8	Methylconiferin	√	√
33	12.502	402.1526	402.1526	0.37	181.0852,242.8844	C18 H26 O10	Acetylcatalpol	√	√
34	14.371	424.2097	424.2097	1.5	211.0616,343.1396,564.7025	C22 H32 O8	Didrovaltratum	√	√
35	14.371	438.2254	438.2254	1.45	362.8406,441.1168,805.9847	C23 H34 O8	Enanderinanin G	√	√

36	64.023	975.9746	975.9746	1.56		C44 H64 O24	Crocin Ⅰ	√	√
37	68.970	813.8245	813.8245	1.20		C38 H54 O19	Crocin Ⅱ	√	√
38	80.717	284.2351	284.2351	1.03	532.1794,1394.3914	C17 H32 O3	Muricatacin	√	√
39	80.717	330.2406	330.2406	0.88	232.9020,154.9741	C18 H34 O5	Sanleng acid	√	√
40	82.13	652.2731	652.2731	1.55	456.8463,554.8148	C32 H44 O14	Crocin Ⅲ	√	√
